# Genetic Assignment Methods for Gaining Insight into the Management of Infectious Disease by Understanding Pathogen, Vector, and Host Movement

**DOI:** 10.1371/journal.ppat.1002013

**Published:** 2011-04-28

**Authors:** Justin V. Remais, Ning Xiao, Adam Akullian, Dongchuan Qiu, David Blair

**Affiliations:** 1 Department of Environmental Health, Rollins School of Public Health, Emory University, Atlanta, Georgia, United States of America; 2 Institute of Parasitic Disease, Sichuan Provincial Center for Disease Control and Prevention, Chengdu, Sichuan, People's Republic of China; 3 Environmental Health Sciences, School of Public Health, University of California, Berkeley, Berkeley, California, United States of America; 4 School of Marine and Tropical Biology, James Cook University, Townsville, Queensland, Australia; University of California San Diego, United States of America

## Abstract

For many pathogens with environmental stages, or those carried by vectors or intermediate hosts, disease transmission is strongly influenced by pathogen, host, and vector movements across complex landscapes, and thus quantitative measures of movement rate and direction can reveal new opportunities for disease management and intervention. Genetic assignment methods are a set of powerful statistical approaches useful for establishing population membership of individuals. Recent theoretical improvements allow these techniques to be used to cost-effectively estimate the magnitude and direction of key movements in infectious disease systems, revealing important ecological and environmental features that facilitate or limit transmission. Here, we review the theory, statistical framework, and molecular markers that underlie assignment methods, and we critically examine recent applications of assignment tests in infectious disease epidemiology. Research directions that capitalize on use of the techniques are discussed, focusing on key parameters needing study for improved understanding of patterns of disease.

## Introduction

For many infectious diseases, transmission is strongly influenced by pathogen, host, and vector migration across complex landscapes [1]. This is especially true for pathogens with environmental stages, or those carried by vectors and intermediate hosts. The spread of rabies, for instance, has been shown to be regulated by rivers that act as barriers to host movement [2], and the onset of diseases such as measles or foot-and-mouth disease is governed in part by human or animal hosts migrating across heterogeneous landscapes [3], [4]. Disease persistence, synchrony, and establishment are known to be modified by host migrations between populations [5]–[9], and thus direct measures of migration rates in real transmission systems are very much needed to optimize disease management and improve intervention campaigns.

Genetic assignment methods can provide such measures; they are a set of powerful statistical approaches that, at their most basic, can be used to establish population membership of individuals. When applied to organisms distributed among spatially distinct, interconnected populations, the techniques can be used to derive quantitative estimates of movement across a network, and determine the degree to which landscape features aid or impede movement. Genetic assignment methods have, for the most part, been limited to applications in ecology and conservation biology. This is despite their utility for estimating the magnitude and direction of key movements in infectious disease systems, where they could reveal important environmental and ecological features that facilitate or limit the spread of disease with important implications for control.

For example, estimates of pathogen transport can be used to design more efficient anthelmintic treatment campaigns for important macroparasites of humans [10], and where environmental change is occurring, estimates of the associated change in migration can aid in the identification of new risks that arise from vectors and hosts moving effectively closer than they have been historically [1]. Genetic assignment tests (ATs) have potential for estimating these pathogen, host, and vector movements, and recent improvements in theory underpinning ATs have increased their utility at fine spatial and temporal scales, while overcoming the cost, time, and scale limitations of traditional approaches such as mark-recapture experiments [11]. Here, we discuss the molecular and statistical methodologies that make possible the application of ATs. We review current applications of ATs in infectious disease epidemiology, and discuss research directions that are positioned to capitalize on use of the techniques. We use the term “migration” to encompass the movement of human hosts, the dispersal of animal hosts and vectors, and the transport of pathogens in environmental media (e.g., flowing water).

## Estimating Migration Rates

While many free-living pathogens, vectors, and intermediate hosts are capable of moving several kilometers, their specific mobilities are rarely estimated or incorporated into efforts to control disease [10], [12]. Historically, ecological migration rates were estimated using direct measures such as mark-recapture and radio tagging, which obviously present limitations when applied to small organisms, large populations with small numbers of migrants, or organisms that are difficult to durably mark [13]. Indirect genetic methods are also available, such as inferring *Nm*, the number of migrants exchanged between populations per generation, using gene flow estimators based on Wright's infinite island model [14], [15]. This approach makes a number of simplifying assumptions, such as assuming symmetrical, constant migration and constant population size, assumptions which were partially relaxed with the development of coalescent-based methods [16].

Coalescent theory describes the statistical properties of gene trees under a standard demographic model (namely the Fisher-Wright model). Present day samples of a non-recombining gene can be seen as lying on a branch of a gene tree rooted at the most recent common ancestor of the sample. Moving backward in time from each branch, genes *coalesce* until the common ancestor is reached, and in this way, present-day samples can be used to infer the past, including past migration among mating populations. Coalescent-based estimates of migration rates, obtained by comparison of allele frequency distributions observed in population samples, assume that all potential source populations have been sampled and that populations have followed relatively simple demographic progressions (constant size or deterministic expansion) while experiencing constant migration [16], [17]. Migration rates obtained in this fashion reflect the effect of migration occurring over long time scales, and do not reflect (i.e., are insensitive to) contemporary changes such as interventions (e.g., vector control) and recent environmental change. ATs, through the combination of highly variable genetic markers with Bayesian statistical methods, allow the estimation of recent migration rates that strongly reflect the influence of contemporary changes.

### Assignment Tests

ATs use multilocus genotypes to identify the source population of individuals that have migrated within the past several generations [18]. Early ATs estimated the probability of an individual's multilocus genotype in relation to the frequency of alleles at different loci in potential source populations. After all sampled individuals were assigned, the migration rate between two populations was estimated by dividing the number of identified migrants by the sample size of the origin population [18]–[20]. A notable recent Bayesian method [21] directly estimates migration rates (and infers inbreeding coefficients and individual migrant ancestries) by detecting the temporary disequilibrium in immigrants' genotypes relative to the population under consideration, while relaxing the assumption that genotypes within subpopulations are in Hardy–Weinberg equilibrium. A related class of clustering methods [19], [22], [23] aims to partition individuals into genetically distinct subpopulations without prior assumptions about population membership; i.e., the methods calculate the probability that each individual genotype originates from one of *K* populations, with *K*, the number of subpopulations, among the inferred parameters.

Bayesian models (also known as fully probabilistic models) provide a convenient means to deal with complex (and inherently stochastic) phenomena that determine the genetic properties of individuals and populations [24]. Like other Bayesian approaches, Bayesian ATs take the position that model parameters and data are random variables with a joint probability distribution specified by a probabilistic model. The model structure and parameters proposed by Wilson and Rannala's [21] notable recent method are described in detail in Text S1. The data and parameters of the inference model implemented in [21] are summarized in Table S1, and Figure S1 shows a probabilistic graphical model indicating the conditional dependencies in [21]. Population assignment is a trivial task if there are fixed differences (no shared alleles) between populations. However, this is rarely the case: typically historical connections, ongoing gene flow, and perhaps convergent evolution lead to the sharing of alleles between populations. Consequently, computationally intensive approaches are required to identify the likely source population of any given individual (see Text S1). Software implementations of Bayesian and maximum likelihood–based methods for inferring migration and population clustering parameters are widely available (Table 1). The extent of population differentiation, the number of individuals that can be sampled, the number of loci, and the specific genetic markers and their polymorphism, all interact in determining the power of any approach [25]. Markers appropriate for ATs are reviewed in detail in Text S2, and different classes of genetic markers and their corresponding advantages and disadvantages are summarized in Table S2.

**Table 1:** Select software packages providing AT functionality, following Excoffier and Heckel [41].

Software	Description	Inference Framework	References
BayesAss+	Provides estimates of recent migration rates between populations using multilocus genotype data	Bayesian, MCMC	[21]
Baps	Assigns individuals to genetic clusters using a partition-based mixture model	Bayesian	[42], [43]
GeneClass	Selects or excludes populations as origins of individuals using multilocus genotype data	Bayesian, likelihood	[44]
Geneland	Detects population subdivisions using multilocus genotype data, accounting for the spatial location of sampled individuals	Bayesian, MCMC	[45]
Structure	Infers the presence of distinct populations, assigns individuals to populations, and identifies migrants using multilocus genotype data	Bayesian, MCMC	[19]

MCMC, Markov chain Monte Carlo.

## Application of ATs in Infectious Disease Systems

Recent infectious disease applications of ATs have estimated pathogen, vector, and host dispersal characteristics in order to explain patterns of transmission and better target control activities. Here, we review four such applications.

### Case 1: Chagas Disease

In the absence of a vaccine or effective theraputics, Chagas disease control is largely dependent on elimination of the vector, members of the genus *Triatoma*, using insecticides. The hematophagous triatomines carry *Trypanosoma cruzi*, the protozoan parasite that causes Chagas disease in much of Latin America. The insects are present in sylvatic and peridomestic populations, with transient and seasonal invasion of homes leading to blood meals and transmission [26]. In the Mexican Yucatán, Dumonteil, Tripet, and colleagues [26] evaluated the genetic structure of *T. dimidiata* to assess dispersal of individuals, better understand domestic infestation, and inform vector control. Insects were sampled from domestic, peridomestic, and sylvatic populations, genotyped at eight microsatellite loci, and analyzed using F statistics and both Bayesian- and likelihood-based ATs [18], [27]. The authors found that *T. dimidiata* is capable of dispersal over large geographic distances in the Yucatán Peninsula (up to 280 km) as suggested by low population differentiation and weak genetic structure. In this case, ATs provided a clearer picture than conventional Fst, allowing for the identification of immigrants even among populations with low genetic differentiation and no detectable correlation between genetic and geographic distance (isolation by distance). ATs indicated that 10%–22% of the insects collected within homes were immigrants from the peridomestic and sylvatic areas. Dispersal was detected in the opposite direction as well, with several insects in peridomestic and sylvatic areas having originated from populations within homes. The ecological basis of genetic structure in this study provided dispersal information that supports pesticide application and refuge removal in peridomestic areas. This zone appears to serve as an important “transit area” between sylvatic and domestic populations, contributing to household reinfestation after control, and largely agreeing with the findings from a small study in Bolivia [28].

### Case 2: *Coccidioides* Species

The *Coccidioides* soil fungi, found in arid zones of the southwestern United States and northwestern Mexico, can cause community-acquired pneumonia and severe disseminated disease (coccidioidomycosis) when inhaled by a vertebrate host [29]. Several western US states have seen dramatic increases in the incidence of coccidioidomycosis (from 2.5 to 8.4 cases per 100,000 in California between 1996 and 2006, and from 21 to 91 cases per 100,000 in Arizona between 1997 and 2006), raising the need for improved surveillance measures [30], [31]. The diagnosis and clinical management of coccidioidomycosis in areas such as New York, where the disease is not endemic, pose unique challenges, and the source of *Coccidioides* infections in these settings is poorly understood. To improve molecular surveillance, identify sources of infection, and allow the early detection and management of outbreaks, Fisher et al. [32] used an AT to assign *Coccidioides* spp. clinical isolates to their populations of origin. The application of ATs to these organisms was complicated by their haploid, rather than diploid, genome, requiring the authors to modify existing AT methods.

More than 160 isolates from eight geographical populations of *Coccidioides immitis* and *Coccidioides posadasii* were genotyped at nine microsatellite loci. Isolates were both clinical and environmental in origin, and spanned the worldwide distribution of *Coccidioides* spp. Sixteen clinical isolates of unknown origin were obtained from patients diagnosed in the nonendemic state of New York. Using a modified AT procedure, 12 of these isolates were assigned to source populations with high probability, most to a source that matched the recent travel history of the patient. Thus, source identification in this nonendemic area was able to detect common-source infections. In two cases, however, travel history did not match assignment, raising questions about whether genetic differentiation was driven by host travel or pathogen dispersal; either an incomplete travel history or exposure to an isolate that had dispersed a great distance could explain the mismatches [32].

### Case 3: Hosts and Vectors of *Yersinia pestis*

*Yersinia pestis*, the bacterium that causes plague, is readily passed between wildlife and humans via flea vectors. In the plains regions of North America, black-tailed prairie dogs (*Cynomys ludovicianus*) live in high-density, communal colonies that favor the spread of plague, making this species an important host for *Y. pestis*. *Oropsylla hirsuta* is a flea very commonly associated with *C. ludovicianus*, and is thought to contribute substantially to *Y. pestis* transmission [33]. Because fleas (and many other ectoparasitic disease vectors) rely on their hosts for dispersal, quantifying host movement can aid in understanding the spread of flea-borne diseases. In a study in the northern US, Jones and Britten [33] investigated the role that prairie dogs play in dispersing fleas infected with *Y. pestis*. The dominant hypothesis in this transmission system, and many others, is that host movements determine vector movements, and thus concordance between host and vector population genetic characteristics would be expected. The study used ATs, among other genetic analyses, to test this hypothesis, sampling 112 prairie dogs from six colonies in north-central Montana and genotyping them at 14 microsatellite loci. At the same time, 84 fleas were collected directly from prairie dog burrows and genotyped at seven microsatellite loci. Genetic structure and variability were analyzed using multiple methods, including the estimation of recent migration rates of prairie dogs and fleas using the Bayesian techniuque described in detail in Text S1 [21].

The authors found that the host and vector differed widely in genetic structure: prairie dog hosts exhibited low intercolony migration (eight of 30 intercolony migration rates showed **m**≥0.05), and the scale of their genetic neighborhood was on the order of a typical colony size. In contrast, the vector was well mixed, showing considerable migration between colony pairs (22 of 30 intercolony migration rates showed **m**≥0.05) and limited colony-level population structure. Because fleas and prairie dog hosts sampled from the same locations show limited concordance in population genetics, it is likely that prairie dogs are not the primary means of *O. hirsuta* dispersal in these colonies. Thus, the authors concluded that other hosts should be considered when responding to plague outbreaks, as *O. hirsuta* occurs on a variety of host species that may be important in dispersing *Y. pestis*–infected fleas [33].

### Case 4: Oral Rabies Vaccination of Racoons

The common raccoon (*Procyon lotor*) is widely distributed throughout North and Central America, and is capable of occupying a broad range of habitats in close proximity to humans. *P. lotor* is also the most frequently reported rabid wildlife species, and is a particularly important carrier of the rabies virus in the mid-Atlantic and northeastern US. Because of the risk of transmission of rabies to humans, the US Department of Agriculture conducts routine oral rabies vaccination programs targeting *P. lotor* and several other important wildlife species. In a large and expensive annual program, recombinant virus vaccine is delivered to *P. lotor* populations in the eastern US in attractive baits. A key question in optimizing these oral rabies vaccine programs is how geographic features (e.g., rivers, mountains, etc.) can be used to better target delivery of baits along important *P. lotor* dispersal corridors, reducing their virus trafficing potential. In a study in southwestern Pennsylvania state, Root, Puskas,and colleagues [34] used ATs to investigate which geographic features, if any, hinder or enhance *P. lotor* dispersal, and thus can be used to improve oral vaccination programs.

Live raccoons were trapped from five study sites distributed along valleys separated by a high elevation ridge; the authors aimed to test the hypothesis that the ridge isolated the populations on either side. DNA from a total of 185 raccoons was genotyped at nine microsatellite loci, and Bayesian clustering [19] and ATs [18] were used to assess the number of genetic clusters and infer the population of origin of *P. lotor* specimens. Specimens from all five study sites were found to compose a single genetic population, and few animals were assigned to their population of origin, with many assigned to sources across the ridge (i.e., sampled from one valley, but assigned to the valley on the opposite side of the ridge; [34]). The results indicate that neither ridge nor valley features in this setting influence *P. lotor* dispersal, as individuals can transcend ridges and can readily traffic virus between (and within) valleys. Thus, ridge and valley features may not be suitable for use in optimizing the geographic placement of oral vaccine baits, despite the finding in other settings that major rivers and mountains may constrain *P. lotor* dispersal [34].

## Discussion

Contemporary movements of hosts can contribute to increased frequency and intensity of malaria epidemics in some regions [35], [36], while transport of free-living pathogen stages can determine the effectiveness of strategies for reducing schistosomiasis infections [10]. Thus, quantifying these movements is of great interest to the study of complex epidemiological systems, and the routine use of ATs for this purpose is anticipated [24].

Among the epidemiological methods that can benefit from ATs are spatial models of infectious disease transmission, which incorporate knowledge of the location, movement rate, and travel direction of hosts, vectors, and pathogens to explain observed patterns of transmission and evaluate intervention options. ATs can provide a quantitative description of migration between populations in transmission models, particularly in the context of network models that explicitly represent the exchange of individuals between populations [1]. Indeed, rigorous quantification of movement between nodes has been called for in network models [4], [37], and ATs offer a powerful alternative to traditional methods (e.g., mark-recapture) that are difficult to apply to these systems.

Challenging epidemiological questions can be addressed by ATs. The source of infection for recombining organisms (as opposed to those organisms where genetic structure is principally clonal) can be determined. As in the *Coccidioides* case, independent loci can be used to estimate the relatedness between isolates and, when combined with travel patterns of infected hosts, assignments can be used to improve surveillance in nonendemic areas, leading to the identification of common source cases that may have otherwise gone undiagnosed [32]. Moreover, ATs can also provide valuable confirmation (or refutation) that a particular host is responsible for the spread of pathogens or vectors [33].

Another key epidemiologcal use for ATs is in assessing the landscape determinants of disease spread. ATs make it possible to formally test previously held beliefs about the role of specific landscape features in governing the mobility of vectors, hosts, and pathogens. Just as valleys and ridges were found not to govern the movement of racoon vectors of rabies [34], conventional wisdom on other landscape determinants of spread can give way to quantitative evidence from ATs. For this to happen, landscape factors must be rigorously characterized and included in the analysis. Simple Euclidean distance between populations has been shown to be inadequate for this purpose [3], [4], and thus alternative (non-Euclidean) distance measures that account for landscape complexity [1] must be employed following the lead of the ecological sciences where much has been learned using this approach [38], [39].

Diffusive processes are ubiquitous in infectious disease transmission [1], and despite limited efforts to quantify these processes in the past, research interest is growing rapidly. The authors of this review are engaged in an application of ATs to *Schistosoma japonicum*, the parasite that causes schistosomiasis in East and Southeast Asia. This organism is subject to transport in the environment via multiple pathways [10]: parasites are carried in advective flows along canals and streams as both larvae and ova; within snail intermediate hosts, parasites are conveyed among and between aquatic and riparian habitats; and for adult worms, human and animal hosts serve as vehicles. ATs provide a powerful means to comprehensively assess the role of these diffusive processes in schistosome transmission, and when combined with landscape data, can offer insights into how anthropogenic change can modify diffusion parameters, thereby influencing transmission. High priority research questions can be addressed, such as which environmental pathways are most influential in maintaining parasite transmission in endemic areas, and which are efficient at spreading the parasite into new regions or among new vulnerable subpopulations?

ATs represent just one analytical avenue in a sophisticated suite of powerful genetic analysis tools available for such epidemiological applications, including other methods for inferring demographic parameters and for identifying genes or genomic regions involved in human diseases [24], [40]. There is diversity even within the set of techniques for estimating migration, and thus, looking forward, comparisons among estimators will be increasingly important, both to validate methods for application to specific hypotheses and to establish confidence in estimates for a particular system.

## Supporting Information

Figure S1 Probabilistic graphical model indicating the conditional dependencies (directed edges) in the Wilson and Rannala [21] method. Nodes represent observed (data; squares) and unobserved (parameters; circles) random variables. The observed variables are the vector of sampled source populations **S** and the matrix of multilocus genotypes of sampled specimens, **X**. Among the unobserved variables (parameters) are the quantities of interest in infectious disease systems, including the interpopulation migration rates in matrix **m** and the specific migrant ancestry of individuals in vector **M**.
(PDF)

Table S1 Data and parameters of the inference model implemented in Wilson and Rannala's [21] Bayesian assignment test.
(PDF)

Table S2 Descriptions of different types of genetic markers and the corresponding advantages and disadvantages when analyzed using assignment tests.
(PDF)

Text S1 Bayesian assignment tests.
(PDF)

Text S2 Genetic markers.
(PDF)
