# Supplementary figures and images for: Genetic Assignment Methods for Gaining Insight into the Management of Infectious Disease by Understanding Pathogen, Vector, and Host Movement
Source: PLoS Pathog. 2011 Apr 28;7(4):e1002013. doi: 10.1371/journal.ppat.1002013 (PMC3084202; doi:10.1371/journal.ppat.1002013)

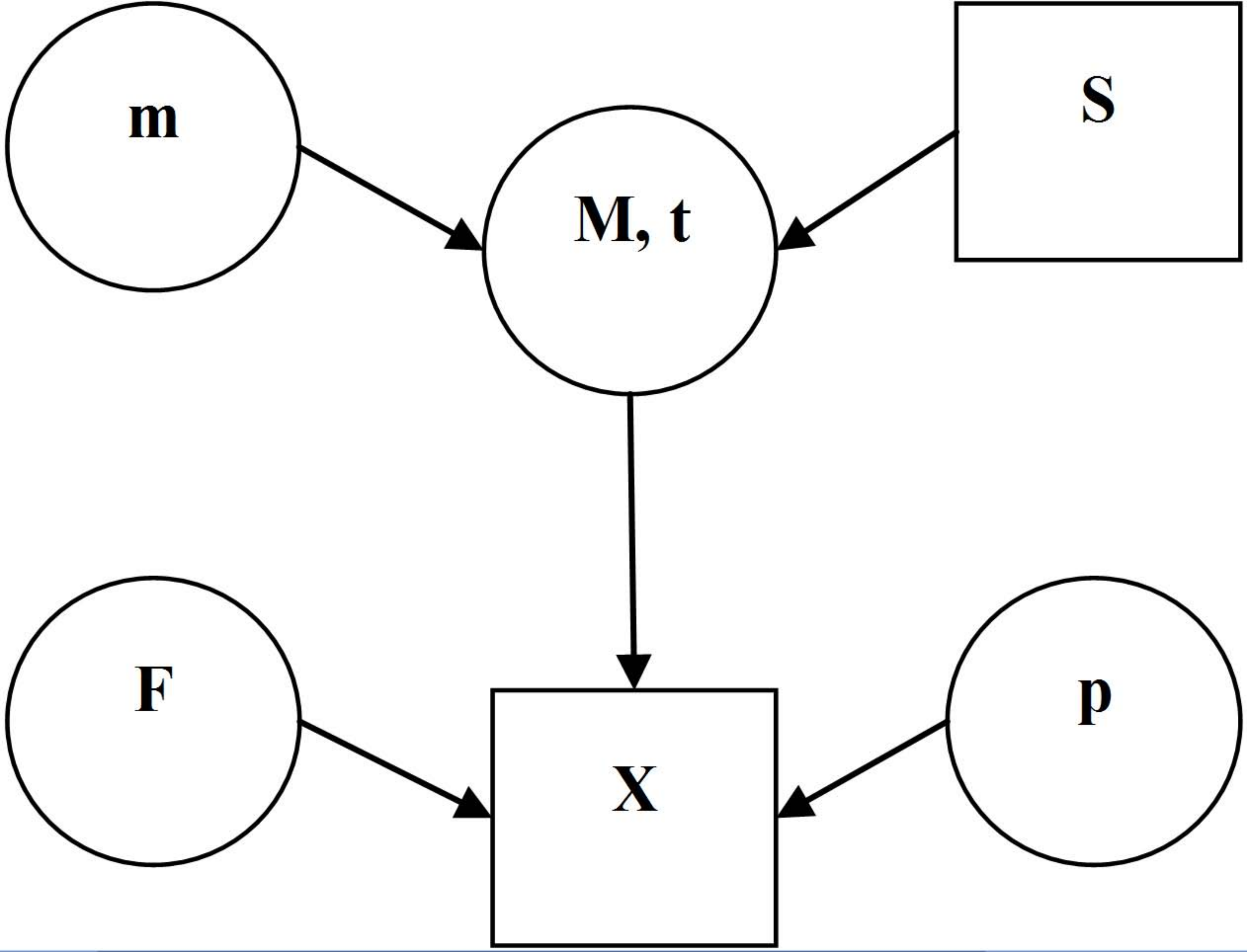

Supplement: Figure S1 — Probabilistic graphical model indicating the conditional dependencies (directed edges) in the Wilson and Rannala [21] method. Nodes represent observed (data; squares) and unobserved (parameters; circles) random variables. The observed variables are the vector of sampled source populations S and the matrix of multilocus genotypes of sampled specimens, X. Among the unobserved variables (parameters) are the quantities of interest in infectious disease systems, including the interpopulation migration rates in matrix m and the specific migrant ancestry of individuals in vector M. (PDF) [file ppat.1002013.s001.pdf]
