# Supplementary material for: Genetic Assignment Methods for Gaining Insight into the Management of Infectious Disease by Understanding Pathogen, Vector, and Host Movement
Source: PLoS Pathog. 2011 Apr 28;7(4):e1002013. doi: 10.1371/journal.ppat.1002013 (PMC3084202; doi:10.1371/journal.ppat.1002013)
Supplement: Table S1 — Data and parameters of the inference model implemented in Wilson and Rannala's [21] Bayesian assignment test. (PDF) [file ppat.1002013.s002.pdf]

Supporting Information – Table S1

Table S1. Data and parameters of the inference model implemented in Wilson and Rannala's [1] Bayesian assignment test.

	Variable	Set notation	Description
Observed variables (data)			
Source populations	**S**	$\{S_h\}$	S_h is the population from which individual *h* was sampled
Genotypes	**X**	$\{X_{hj}\}$	X_{hj} is the genotype of individual *h* at locus *j*
Unobserved variables (parameters)			
Migration rates	**m**	$\{m_{lq}\}$	m_{lq} is fraction of individuals in population *q* that migrated from population *l*
Migrant ancestry	**M**	$\{M_h\}$	M_h is the source of migrant ancestry of individual *h*
Time of migration	**t**	$\{t_h\}$	t_h is the generation at which a migrant ancestor of individual *h* arrived
Population allele frequencies	**P**	$\{p_{ijl}\}$	p_{ijl} is the frequency of allele *i* at locus *j* in population *l*
Inbreeding coefficient	**F**	$\{F_l\}$	F_l is the inbreeding coefficient for population *l*

1. Wilson, G. and B. Rannala, *Bayesian Inference of Recent Migration Rates Using Multilocus Genotypes.* Genetics, 2003. **163**: p. 1177-1191.
