# Supplementary material for: Genetic Assignment Methods for Gaining Insight into the Management of Infectious Disease by Understanding Pathogen, Vector, and Host Movement
Source: PLoS Pathog. 2011 Apr 28;7(4):e1002013. doi: 10.1371/journal.ppat.1002013 (PMC3084202; doi:10.1371/journal.ppat.1002013)
Supplement: Table S2 — Descriptions of different types of genetic markers and the corresponding advantages and disadvantages when analyzed using assignment tests. (PDF) [file ppat.1002013.s003.pdf]

Supporting Information – Table S2

Table S2. Descriptions of different types of genetic markers and the corresponding advantages and disadvantages when analyzed using assignment tests.

Genetic Marker	Description	Advantages	Disadvantages
Allozymes	Different forms of the same enzyme coded at the same locus	Straightforward and relatively cheap.	Low levels of polymorphism. Require frozen tissue
Microsatellites	Tandem repeats of 1-6 base pairs of DNA, flanked by unique sequence for primer design	Highly polymorphic. Easily reproducible. Amenable to many forms of analysis.	Relatively high cost of development (need prior sequence data) and of genotyping. Requires calibration if different labs work on the same organism.
Single nucleotide polymorphisms (SNPs)	Variants at a single base position.	Abundant in genome. Can be scored from poor-quality DNA. Platforms exist for cheap automated genotyping at many loci	Limited polymorphism (generally bi-allelic). Relatively high cost of development (need prior sequence data). Need to genotype at many loci. No calibration required between labs.
Amplified Fragment Length Polymorphisms (AFLPs)	Differing locations of restriction sites produce DNA fragments of different lengths in PCR	Inexpensive. Generally high levels of polymorphism and of repeatability. No prior sequence data required.	Cannot assign any band to a particular locus, thus limiting analysis. Optimization can be tedious.
