# Supplementary material for: Genetic Assignment Methods for Gaining Insight into the Management of Infectious Disease by Understanding Pathogen, Vector, and Host Movement
Source: PLoS Pathog. 2011 Apr 28;7(4):e1002013. doi: 10.1371/journal.ppat.1002013 (PMC3084202; doi:10.1371/journal.ppat.1002013)
Supplement: Text S1 — Bayesian assignment tests. (PDF) [file ppat.1002013.s004.pdf]

Supporting Information - Text S1

Bayesian assignment tests

Like other Bayesian approaches, Bayesian assignment tests take the position that model parameters and data are random variables with a joint probability distribution specified by a probabilistic model. In the model proposed by Wilson & Rannala's [1] assignment method (summarized in Figure S1 and Table S1), the observed variables (the data) are the vector of sampled source populations **S** and the matrix of multilocus genotypes of sampled specimens, **X.** Among the unobserved variables (the parameters) are the quantities of interest in infectious disease systems, including the interpopulation migration rates in matrix **m** and the specific migrant ancestry of individuals in vector **M**.

The goal of the Bayesian method is to calculate the posterior distribution of these parameters (the conditional distribution of the parameters given the data). The procedure involves making probability statements about the parameters before observing the data, assigning so-called 'prior' probability distributions to each. For instance, the probability of observing **M** and **t** given **m**, $\Pr(\mathbf{M},\mathbf{t}|\mathbf{m})$, follows a multinomial distribution derived from expected migrant proportions assuming negligible genetic drift over two or three generations. The likelihood of the data is specified as the probability of the observed genotypes given the parameters, $\Pr(\mathbf{X}|\mathbf{S};\mathbf{M},\mathbf{t},\mathbf{F},\mathbf{p})$, and Bayes theorem is applied to obtain the joint posterior probability distribution, $f(\mathbf{m},\mathbf{M},\mathbf{t},\mathbf{F},\mathbf{p}|\mathbf{X},\mathbf{S})$, of model parameters [1]:

$$f(\mathbf{m},\mathbf{M},\mathbf{t},\mathbf{F},\mathbf{p}|\mathbf{X},\mathbf{S}) = \frac{\Pr(\mathbf{X}|\mathbf{S};\mathbf{M},\mathbf{t},\mathbf{F},\mathbf{p}) \times \Pr(\mathbf{M},\mathbf{t}|\mathbf{m})\, f_{\mathbf{m}}(\mathbf{m})\, f_{\mathbf{p}}(\mathbf{p})\, f_{\mathbf{F}}(\mathbf{F})}{\Pr(\mathbf{X}|\mathbf{S})} \qquad (0.1)$$

where priors on **m**, **p** and **F** are given by $f_{\mathbf{m}}(\mathbf{m})$, $f_{\mathbf{p}}(\mathbf{p})$ and $f_{\mathbf{F}}(\mathbf{F})$, respectively, and $\Pr(\mathbf{X}|\mathbf{S})$ is the so-called marginal likelihood of the data, the probability of the data irrespective of the parameter values. Essentially, data are combined with prior knowledge to obtain posterior parameter estimates, distinguishing Bayesian techniques from maximum-likelihood methods that do not incorporate prior information, and are based entirely on finding parameters that maximize the probability of the data given the parameters.

Probability models in population genetics often contain interrelated parameters that are constrained to a particular set of values, providing useful priors to initiate the Bayesian procedure.

Population assignment is a trivial task if there are fixed differences (no shared alleles) between populations. However, this is rarely the case: typically historical connections, ongoing gene flow and perhaps convergent evolution lead to the sharing of alleles between populations. Consequently, computationally intensive approaches are required to identify the likely source population of any given individual. The computation of $f(\mathbf{m},\mathbf{M},\mathbf{t},\mathbf{F},\mathbf{p}|\mathbf{X},\mathbf{S})$ is essentially a high-dimensional integration problem, requiring numerical methods such as Monte Carlo Markov Chain (MCMC). MCMC methods allow the generation of observations from complex probability distributions, and thus provide a means of sampling from posterior probability distributions and making inferences [for detailed discussion, see 2, 3, 4]. MCMC methods for inferring the value of migration (e.g. m) and population clustering (e.g. K) parameters have been implemented in a number of software platforms. The extent of population differentiation, the number of individuals that can be sampled, the number of loci, and the specific genetic markers and their polymorphism, all interact in determining the power of any test [4].

References

1. Wilson, G. and B. Rannala, *Bayesian Inference of Recent Migration Rates Using Multilocus Genotypes.* Genetics, 2003. **163**: p. 1177-1191.
2. Brooks, S.P., *Markov chain Monte Carlo method and its application.* Journal of the Royal Statistical Society Series D-the Statistician, 1998. **47**(1): p. 69-100.
3. Besag, J., et al., *Bayesian Computation and Stochastic-Systems.* Statistical Science, 1995. **10**(1): p. 3-41.
4. Faubet, P.W., Robin S.; Gaggiotti, Oscar E., *Evaluating the performance of a multilocus Bayesian method for the estimation of migration rates.* Molecular Ecology, 2007. **16**: p. 18.
