# Supplementary material for: Genetic Assignment Methods for Gaining Insight into the Management of Infectious Disease by Understanding Pathogen, Vector, and Host Movement
Source: PLoS Pathog. 2011 Apr 28;7(4):e1002013. doi: 10.1371/journal.ppat.1002013 (PMC3084202; doi:10.1371/journal.ppat.1002013)
Supplement: Text S2 — Genetic markers. (PDF) [file ppat.1002013.s005.pdf]

Supporting Information - Text S2

Genetic markers

Markers appropriate for assignment tests (Table S2) fall into two main classes, co-dominant and dominant. Co-dominant markers, such as allozymes, microsatellites and single nucleotide polymorphisms (SNPs), are most commonly used. In this class of markers, all alleles present at any particular locus in an individual organism can be detected and scored. Of the co-dominant markers, allozymes are the cheapest but typically have the least resolution (low levels of polymorphism and few alleles per locus). Allozymes are allelic variants of enzymes that can be extracted from tissues in aqueous solution. The complex mixture in the extraction can be separated electrophoretically by size, shape and charge, and the location of the desired enzyme in the gel demonstrated by specific staining [1]. Allozymes are no longer commonly used in population studies despite their demonstrated utility [2].

The remaining co-dominant markers depend on the polymerase chain reaction (PCR) to amplify DNA from individual loci. Microsatellites, tandem repeats of short (typically 2-6bp) motifs and flanked by unique-sequence regions that can be exploited for primer design, are currently the most popular of these. Repeat motifs typical of microsatellites are prone to replication errors, thus generating a large number of alleles in the population gene pool. Characterization of microsatellites for a previously unstudied species has until recently been a slow and expensive task involving the construction and probing of genomic libraries. However, draft genomes are accumulating for a great variety of species and can be searched for microsatellite loci *in silico* [e.g. 3]. The advent of next-generation sequencing also offers a rapid and cost-effective way of finding microsatellites. A single Roche 454 pyrosequencing run will generate such a volume of random genomic data that many microsatellite loci are certain to be included [e.g. 4]. For microsatellites, various methods have been proposed to assess which loci provide the greatest discriminatory power [critiqued by 5, 6].

Single nucleotide polymorphisms (SNPs) form another group of co-dominant markers. A SNP is a variant occurring at a single base position and is the most common form of

polymorphism in the genome [7]. SNPs can be found by study of genomic resources and by comparisons of numerous individuals to detect variant sites. They are normally bi-allelic, which limits their discriminatory power and requires larger numbers of loci to be genotyped than is required for microsatellites [8, 9]. However, there are many potential benefits to using SNPs instead of or in addition to microsatellites [9-11]. Platforms for automated and rapid genotyping of large numbers of SNPs are available, and technical advances in this area are continuing. SNPs can be genotyped more readily than microsatellites from poor quality and ancient DNA samples [12] and are more easily compared between studies.

The second main class, dominant genetic markers, includes amplified fragment length polymorphisms (AFLPs) and random amplified polymorphic DNA (RAPD), in which multiple bands are generated cheaply and rapidly by PCR. These bands cannot be assigned to a particular genomic location and each is generally scored simply as present or absent. Disadvantages are that departure from Hardy-Weinberg equilibrium cannot be assessed and homology is assumed based on band length. A major advantage is that no prior knowledge of genomic sequence is required. AFLP markers [13] are relative late-comers to assignment tests, the first use being by Duchesne and Bernatchez [14]. In generating AFLP data, the first step is digestion of genomic DNA with a restriction enzyme. Adaptors are ligated to the sticky ends of the fragments, and selective primers are then used in PCR to generate bands. The selective primers include the sequence of the adaptor and restriction site used, as well as several nucleotides at the 3' end that enforce annealing only with those fragments possessing these nucleotides. Following PCR, fragments are separated electrophoretically. Campbell, Duchesne et al. [15] used presence or absence of bands in a study on the population-discrimination power of AFLPs relative to microsatellites. They concluded that, for weakly differentiated populations, AFLPs could be the superior form of marker. Falush, Stephens et al. [16] have described the use of AFLP data in the program Structure.

References

1. Richardson, B., P. Baverstock, and M. Adams, *Allozyme electrophoresis: a handbook for animal systematics and population studies*. 1986, Sydney: Academic Press.
2. Conte, R., et al., *Genetic structure and mating system of Euterpe edulis Mart. Populations: a comparative analysis using microsatellite and allozyme markers.* J Hered, 2008. **99**(5): p. 476-82.
3. Yin, M., et al., *Multiple near-identical genotypes of Schistosoma japonicum can occur in snails and have implications for population-genetic analyses.* Int J Parasitol, 2008. **38**(14): p. 1681-91.
4. Perry, J.C. and L. Rowe, *Rapid Microsatellite Development for Water Striders by Next-Generation Sequencing.* J Hered, 2010.
5. Waples, R.S., *High-grading bias: subtle problems with assessing power of selected subsets of loci for population assignment.* Mol Ecol, 2010. **19**(13): p. 2599-601.
6. Anderson, E.C., *Assessing the power of informative subsets of loci for population assignment: standard methods are upwardly biased.* Molecular Ecology Resources, 2010. **10**: p. 701-710.
7. Seddon, J.M., et al., *SNPs in ecological and conservation studies: a test in the Scandinavian wolf population.* Mol Ecol, 2005. **14**(2): p. 503-11.
8. Morin, P., K. Martien, and B. Taylor, *Assessing statistical power of SNPs for population structure and conservation studies.* Molecular Ecology Resources, 2009. **9**(1): p. 66-73.
9. Morin, P., et al., *Characterization of 42 single nucleotide polymorphism (SNP) markers for the bowhead whale (Balaena mysticetus) for use in discriminating populations.* Marine Mammal Science, 2010. **26**(3): p. 716-32.
10. Beacham, T., B. McIntosh, and C. Wallace, *A comparison of stock and individual identification for sockeye salmon (Oncorhynchus nerka) in British Columbia provided by microsatellites and single nucleotide polymorphisms.* Canadian Journal of Fisheries and Aquatic Sciences, 2010. **67**(8): p. 1274-90.
11. Glover, K.A., et al., *A comparison of SNP and STR loci for delineating population structure and performing individual genetic assignment.* BMC Genet, 2010. **11**: p. 2.
12. Morin, P. and M. McCarthy, *Highly accurate SNP genotyping from historical and low quality samples.* Molecular Ecology Notes, 2007. **7**: p. 937–946.
13. Vos, P., et al., *AFLP: a new technique for DNA fingerprinting.* Nucleic Acids Res, 1995. **23**(21): p. 4407-14.
14. Duchesne, P. and L. Bernatchez, *AFLPOP: a computer program for simulated and real population allocation, based on AFLP data.* Molecular Ecology Notes, 2002. **2**: p. 380-383.
15. Campbell, D., P. Duchesne, and L. Bernatchez, *AFLP utility for population assignment studies: analytical investigation and empirical comparison with microsatellites.* Mol Ecol, 2003. **12**(7): p. 1979-91.
16. Falush, D., M. Stephens, and J.K. Pritchard, *Inference of population structure using multilocus genotype data: dominant markers and null alleles.* Molecular Ecology Notes, 2007. **7**(4): p. 574-578.
